# Performance, Failures, and Oversight of a Large Language Model Agent for Clinical Data Analysis: Evaluation Study

**DOI:** 10.2196/99597

**Published:** 2026-09-08

**Authors:** Yilan Wu, Dun Jack Fu, Yukun Zhou, Siegfried K Wagner, Pearse A Keane

**Affiliations:** 1 Institute of Ophthalmology, University College London, 162 City Rd, London, England, EC1V2PD, United Kingdom, 1 7482213973; 2 NIHR Biomedical Research Centre at Moorfields Eye Hospital NHS Foundation Trust, London, United Kingdom; 3 Hawkes Institute, University College London, London, United Kingdom

**Keywords:** AI, data analysis, large language model, ophthalmology, survival analysis

## Abstract

**Background:**

Large language model (LLM) agents capable of generating and executing statistical code from natural language may broaden access to clinical data analysis, yet which pipeline stages they perform reliably and which require expert oversight remain poorly defined.

**Objective:**

This study aimed to evaluate the performance and systematic failure modes of an LLM agent across 5 stages of a clinical data analysis workflow.

**Methods:**

The publicly available dataset and R script (R Foundation for Statistical Computing) were drawn from a previously published study of 12-year outcomes in 7802 patients with eyes with neovascular age-related macular degeneration at Moorfields Eye Hospital. Participants were evaluated using an LLM agent (Claude; Anthropic) across 3 interaction modes (Chat, Code, and Cowork). It was asked to perform 3 levels of data analysis practice: prompt A, to generate research questions from raw data only; prompt B, to develop a statistical analysis plan (SAP) from a high-level clinical objective, then execute it; and prompt C, to execute an analysis given an investigator-drafted SAP. Each was replicated 3 times (27 total runs). Qualitative evaluation of research question thematic coverage (prompt A), SAP completeness against a reference checklist (prompt B), and evaluation of execution outputs against validated reference values and of result text and narrative summaries against execution logs (prompts B and C) was conducted.

**Results:**

The agent generated 18 clinically grounded questions spanning 7 domains; Cowork mode uniquely reached 3 thematic areas requiring data-driven methods. All 9 SAPs correctly identified the statistical framework. Kaplan-Meier estimates were near-identical across 17 completed runs. Systematic execution errors emerged: SAP quality did not predict code correctness, and within-mode errors propagated identically across independent repetitions. Result text accurately reflected execution logs in nearly all runs, though unit propagation and an undisclosed postcrash rerun were identified. Of 17 narrative summaries, 8 were fully satisfactory; 2 runs produced clinically meaningful errors.

**Conclusions:**

LLM agents perform reliably for question generation and SAP drafting but require expert verification of formula composition, cohort boundary logic, and concordance computation before results are reported. Using an ophthalmology dataset as a controlled testbed, this study develops and applies an evaluation framework whose lessons are likely applicable across clinical specialties.

## Introduction

Large language model (LLM)-powered tools capable of generating and executing statistical code have begun to enter clinical research workflows [1-4]. Clinical data analysis requires substantial statistical and epidemiological expertise that may not be uniformly available to clinician-researchers [5-7]. LLM-powered agents offer a compelling model: a researcher states a research question, and the agent drafts a statistical plan and runs the analysis [8,9].

Despite this promise, a systematic evaluation of where LLM agents succeed and where they fail in clinical research workflows is lacking. In the field of clinical data analysis, which requires expertise to enter, it is possible for an agent to produce plausible-looking outputs while harboring methodological errors that are invisible to a nonexpert reviewer [10]. Existing benchmarks focus on code correctness for isolated tasks or on multiple-choice medical knowledge [1,11], without examining the full chain from question formulation through protocol design to statistical execution on real clinical data.

This pilot study evaluated an LLM agent across 5 stages of clinical data analysis, using a publicly released dataset and validated R (R Foundation for Statistical Computing) script from a previously published JAMA (Journal of the American Medical Association) Ophthalmology study [12] as a controlled benchmark. Three interaction modes spanning a spectrum from conversational use (Chat) to fully autonomous local execution (Code and Cowork) were compared across 3 prompt levels to identify systematic error patterns and to inform practical deployment guidance. Although the benchmark is grounded in ophthalmology, the data analysis workflow evaluated in this study (clinical question generation, statistical analysis plan [SAP] drafting, analysis execution, and result summarization and manuscript writing) is common to observational research across medical specialties. Accordingly, we identify and summarize generalizable error patterns and an evaluation framework for assessing future LLM agent releases in clinical data analysis more broadly.

The primary objective of this study was to evaluate the reliability, failure modes, and required human oversight of LLM agents across 5 stages of the clinical data analysis pipeline: research question generation, SAP formulation, data preprocessing and cohort boundary logic, statistical execution, and narrative clinical reporting. We hypothesized that while LLM agents could generate clinically relevant research questions and technically compliant statistical plans, their autonomous code execution would introduce silent, plausible-looking logical errors (such as cohort boundary mismatches, mathematical tie violations, or statistical assumption check omissions) that require mandatory expert biostatistical review and cannot be detected from runtime success or final numerical summaries alone.

## Methods

### Dataset and Reference Standard

This study used the publicly available dataset and reference R script from Fu et al [12]. The original study examined 12-year longitudinal anti–vascular endothelial growth factor treatment outcomes for neovascular age-related macular degeneration in 7802 eyes (118,255 clinic visits at Moorfields Eye Hospital, October 2008-February 2020) using Cox proportional hazards (PHs) models and Kaplan-Meier (KM) estimators and released both the dataset and script publicly alongside the publication. Three time-to-event outcomes were benchmarked: (O1) time to visual acuity (VA) at least 70 Early Treatment Diabetic Retinopathy Study (ETDRS) letters, (O2) duration of sustained VA at least 70 letters, and (O3) time to VA decline to 35 or fewer letters. Quantitative outputs (cohort sizes, KM medians, hazard ratios [HRs], and concordance statistics) served as the evaluation benchmark.

### Ethical Considerations

This study did not involve human participants, clinical trials, or the collection or analysis of primary patient data. All experiments were conducted using a publicly available, fully deidentified database [12] and a reference R script released under a Creative Commons Zero license. Consequently, in accordance with the UK Health Research Authority (HRA) and National Health Service (NHS) research ethics guidelines [13], as well as the institutional policies of Moorfields Eye Hospital and University College London, this study was exempt from institutional ethics review board assessment. This study was reported in accordance with the DECIDE-AI (Developmental and Exploratory Clinical Investigations of Decision Support Systems Driven by AI) reporting guidelines [14].

### Prompt Framework

Three prompts were developed to ask the agent to conduct different steps of the data analysis pipeline using different predefined information.

**Figure 1.**
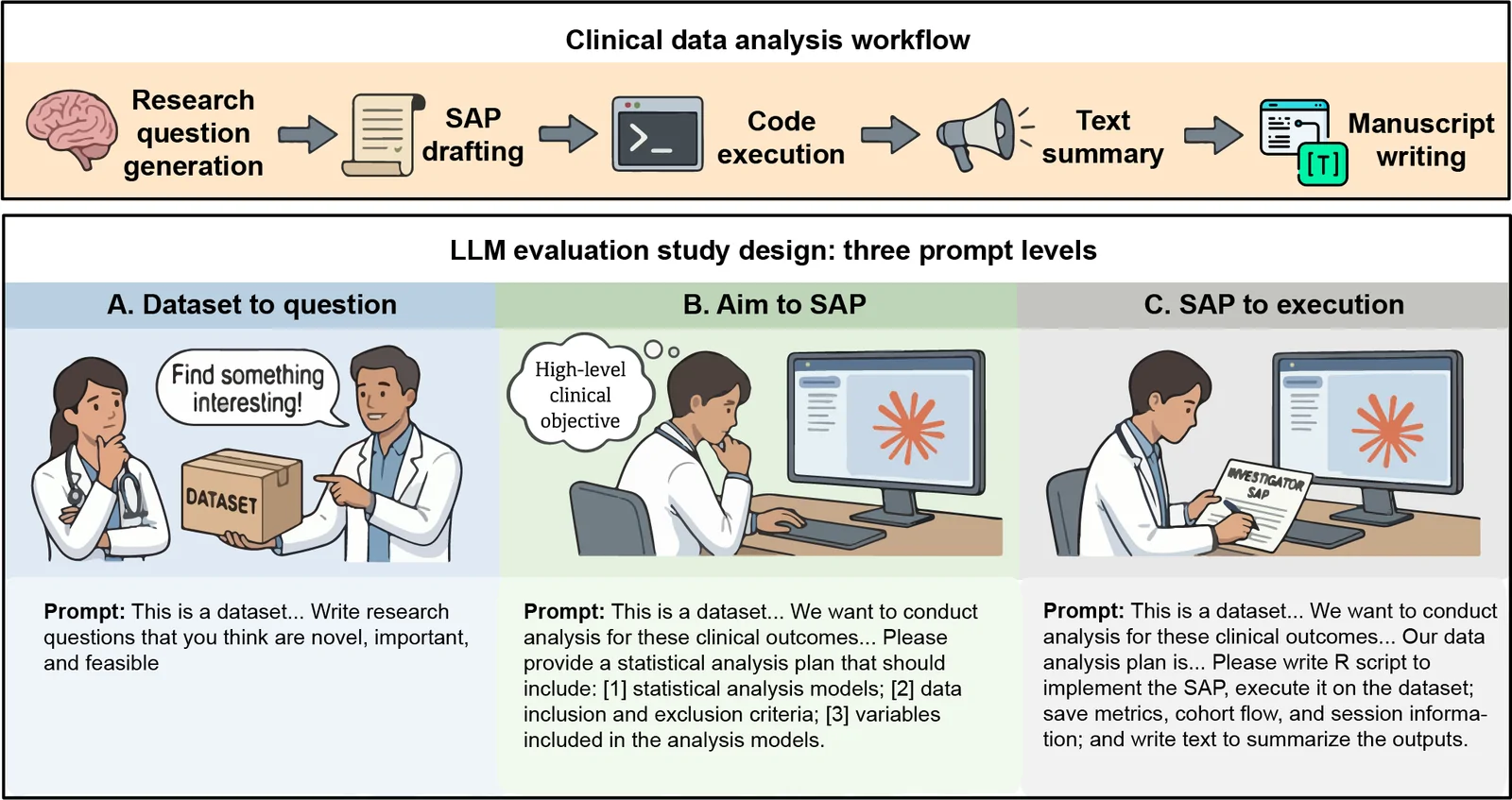
Study design overview. Different levels of prompts can be interpreted as different clinicians’ mental labor and input levels. Prompt A, from dataset to question, simulates the workflow when a junior researcher is given a dataset by their mentor but without a specific project, so they must first identify a scientific question. Prompt B, from aim to statistical analysis plan (SAP), represents when a junior researcher only receives high-level guidance and must develop a detailed protocol independently. Prompt C, with specific SAP input, simulates when the junior researcher already has a detailed protocol and can directly proceed with execution. LLM: large language model.

Prompt A is designed for research question generation, where the agent received the dataset and data dictionary [15] and was asked to propose 2 novel, clinically meaningful, and analytically feasible research questions. Prompt B is designed for SAP development and execution, where the agent received 3 outcome definitions as high-level clinical objectives, independently drafted a complete SAP, and then implemented and executed it. Prompt C is designed for guided execution, where the agent received an SAP and implemented it (Figure 1; Method S2 in Multimedia Appendix 1).

The outcome definitions for prompt B were extracted from the published abstract of the original publication [12], and the prompt C SAP was drafted based on the Methods section, specifying model family, covariates, cohort definitions, and reporting requirements from the original publication [12]. Only the prompts, dataset, and data dictionary [14] were provided to the agent, and no reference to the original paper or code was provided.

### Agent Platform and Interaction Modes

All experiments used Claude (Anthropic) in 3 modes: Chat (conversational; no autonomous file-system or script execution; free tier), Code (writes files and executes R or Python code locally; paid subscription), and Cowork (highest autonomy; may independently choose programming language and analytical strategy; paid subscription). Each mode-prompt combination was replicated 3 times in fully independent sessions, yielding 27 total runs. The runs were coded as A/B/C (prompt)_Chat/Code/Cowork (mode)_1/2/3 (replicate number).

All experiments were done by Claude Sonnet (version 4.6). Claude for Mac was accessed from February 27, 2026, to July 04, 2026, to run the experiments. Zero-shot model memory probing was run to avoid model contamination.

### Evaluation Framework

#### Research Question Quality (Prompt A)

All 18 questions were evaluated qualitatively. Each question was assigned to a thematic group based on the primary clinical construct addressed by the question. Thematic groups were derived inductively from the question set by a clinician-researcher reviewer and cross-checked against the data dictionary [15]. LLM agent–generated questions were rated for novelty, clinical importance, and analytical feasibility on a 5-point Likert scale (1=“lowest“ to 5=“highest”). Novelty and importance were evaluated against existing publications. Feasibility was evaluated against the available covariates in the released database. Two graders were involved in the assessment, one with 3 years of clinical and ophthalmology research experience, and the other with over 10 years of clinical and ophthalmology research experience.

#### SAP Quality (Prompt B)

Each SAP was assessed against a reference checklist from the Methods section of the original publication, covering statistical model specification, covariate completeness, cohort definitions, and reporting requirements. The generated R scripts were validated by comparing each run against the reference standard: first, for correctness (computational execution), we verified whether the generated scripts executed successfully to completion without runtime crashes or programming bugs. This was validated as the final result, as the agent might internally debug and self-correct in the process to complete a targeted run. Then, for statistical validity (numerical accuracy), we manually audited and compared the resulting quantitative outputs against the validated reference values of the original reference publication. Lastly, for implementation (code logic and variable selection), for any run that exhibited a numerical deviation from the reference standard, we manually reviewed the lines of code in each script to verify whether the data preprocessing, cohort-filtering boundaries, and regression formulas correctly reflected the SAP. The manual validation was conducted by a researcher with 6 years of clinical data analysis experience, and confirmed by the author of the reference study, who performed the ground truth data analysis.

#### Analysis Execution Quality (Prompts B and C)

Scripts were assessed for Cox formula composition, cohort boundary logic, and counting-process implementation; quantitative outputs were compared with reference values.

#### Script Output-to-Result Text Fidelity (Prompts B and C):

The text summary was asked to directly report the key statistical analysis results, including, but not limited to, the sample size, baseline descriptions, methods, variables, and results for each analysis conducted (Method S1 in Multimedia Appendix 1). Each run’s result summary was compared line-by-line against its execution log for numerical accuracy and potential hallucinations.

#### Manuscript Writing Quality (Prompts B and C)

Manuscripts were asked to use the computed output to get high-level conclusions in a manuscript-like narrative style, without specific length or style guidance (Methods S1 and S2 in Multimedia Appendix 1). Each manuscript was evaluated by a clinician reviewer against the reference paper across three criteria: (1) interpretation, about the appropriate use of associative language and the correct direction and magnitude of findings; (2) clinical implications, about logical derivation from results, proportionate conclusions, and appropriate observational caveats; and (3) reporting alignment, about the correspondence with the key findings of the reference paper without omission or invention. Each criterion was rated as satisfactory, minor issue, or significant issue.

#### Error Pattern Taxonomy

Failure modes across all pipeline stages were aggregated inductively from the qualitative evaluations above. Repeated errors were classified according to the mechanisms, in order to develop a practical taxonomy for guiding expert verification of LLM-generated analyses.

## Results

### Research Question Quality (Prompt A)

All 9 prompt A runs generated 2 questions each (18 total; 100% completion rate). Thematic analysis identified 7 distinct clinical domains across the 18 questions (Table S1 in Multimedia Appendix 1). These themes were as follows: induction phase completeness and long-term VA (group-a, n=6, 33% questions); injection interval and variability (group-b, n=5, 28% questions); early VA response as a prognostic biomarker (group-c, n=3, 17% questions); treatment burden and maintenance phenotype (group-d, n=1, 6% question); latent VA trajectory phenotyping (group-e, n=1, 6% question); withheld injection visits and VA decline (group-f, n=1, 6% question); and ethnic disparities in VA outcomes (group-g, n=2, 11% questions).

Thematic coverage was unequal and mode-dependent. Group-a alone accounted for 6 of 18 (33%) questions, reflecting consistent agent anchoring on the most structurally prominent binary variable in the dataset (loaded or not loaded). Chat and Code modes generated questions exclusively in groups a to c. Cowork was the only mode to reach group-e (latent trajectory phenotyping), group-f (withheld injection adherence), and groups-g (ethnic disparity mediation)—3 thematic domains not generated by Chat or Code in any of the 6 combined runs.

Mean scores were 3.0 (SD 0.69) for novelty, 4.2 (SD 0.43) for importance, and 3.5 (SD 0.92) for feasibility on a 5-point scale. Thematic groups with higher novelty (such as withheld injections or ethnic disparities) suffer from low feasibility (2.00) due to dataset variable limitations (missing access or mediation variables or noninjection visit coding). Conversely, routine questions like induction completeness are highly feasible (4.40) but have low novelty (2.80).

### SAP Quality (Prompt B)

All 9 SAPs correctly identified Cox PHs and KM estimation and specified the main covariate domains (Table S2 in Multimedia Appendix 1). Chat mode produced the most specific technical language: B_Chat_1 explicitly specified that VA be scaled per 5 ETDRS letters and that injection count must be excluded from the Cox formula, which was the most methodologically precise SAP in the study. However, B_Chat_2 introduced ambiguity with the permissive phrase “may be used” for injection count. Code mode SAPs were nearly identical across all 3 repetitions but systematically underspecified VA scaling and injection count handling, indicating within-mode stereotyping. Cowork mode showed the widest variation: B_Cowork_2 produced the most detailed SAP overall, while B_Cowork_1 produced the least specific SAP.

Beyond the core of KM and Cox PH, the 9 SAPs proposed a broad set of ancillary methods (Table S3 in Multimedia Appendix 1). Competing risks modeling (Fine-Gray subdistribution hazard) was the most consistently proposed extension, appearing in all 9 SAPs and most often for Outcome 3 (time to VA≤35), with treatment discontinuation as the competing event. Landmark analysis was proposed by all 9 SAPs to address immortal-time and selection bias in Outcome 2. Code-mode SAPs were the most conservative, consistently proposing Fine-Gray, landmark, and restricted mean survival time but rarely venturing further; Chat mode was the most expansive; Cowork occupied a middle ground with multistate and frailty models.

### Analysis Execution Quality (Prompts B and C)

KM median estimates were highly consistent across all conditions (O1: 2.03‐2.04 y; O2: 0.54 y; O3: 8.65‐8.66 y), suggesting that KM estimation may be a dependable pipeline component (Table 1).

**Table 1.** Analysis execution quality by run.

Run	Cohort O1	Injection count in Cox	HR^a^ (O1)	Concordance (O1)	Key execution finding
Reference	5978	✗^b^ excluded	1.434	0.749	
B_Chat_1	5794 (−184)	✗ crash	✗ crash	0.700 (rerun)	VA^c^ entered categorically; crashed; result summary reflects corrected rerun
B_Chat_2	5794 (−184)	✗ absent	▲^d^ 1.075	0.749 ✓^e^	Injection count absent (hedged SAP)^f^; VA per letter
B_Chat_3	5794 (−184)	▲^g^	▲^d^ 1.075	0.749 ✓	Partial TDC^h^ matches partial SAP
B_Code_1	5794 (−184)	▲^g^	▲^d^ 1.075	0.749 ✓	VA per letter; within-mode stereotyping
B_Code_2	5794 (−184)	▲^g^	▲^d^ 1.075	0.749 ✓	Identical to B_Code_3
B_Code_3	5794 (−184)	▲^g^	▲^d^ 1.075	0.749 ✓	Identical to B_Code_2
B_Cowork_1	5978 ✓	✗ absent (Python)	✓ 1.427	—^i^	Lowest SAP specificity; best cohort
B_Cowork_2	5978 ✓	▲^g^	✗ 1.329	0.668	Best SAP; injection count included despite SAP exclusion
B_Cowork_3	5794 (−184)	▲^g^	✓ 1.427	—	Moderate SAP; consistent implementation
C_Chat_1	5978 ✓	▲^g^	✗ 1.260	—	Custom CP; injection count in formula biases HR
C_Chat_2	5978 ✓	▲^g^	✗ 1.219	—	tmerge; −15% HR bias
C_Chat_3	5978 ✓	▲^g^	✓∼1.430	—	HR unaffected in this run
C_Code_1	5978 ✓	▲^g^	✗ 1.264	—	Custom CP; injection count biases HR
C_Code_2	5978 ✓	▲^g^	✓ 1.436	0.753 ✓	Most accurate run
C_Code_3	5978 ✓	▲^g^	✓∼1.430	—	Consistent with C_Code_2
C_Cowork_1	5978 ✓	▲^g^	✓ 1.433	—	Row count matches reference; HR accurate
C_Cowork_2	5809 ✗	▲^g^	✗ 1.261	✗ 0.867	Python interval over-splitting; concordance inflated
C_Cowork_3	—	—	—	—	Complete execution failure

a HR: hazard ratio.

b Incorrect answer.

c VA: visual acuity.

d The agent reported the HR per individual Early Treatment Diabetic Retinopathy Study (ETDRS) letter rather than the standard 5-letter standard.

e Correct answer.

f SAP: statistical analysis plan.

g The agents partially implemented the requirement by recognizing that “injection count” was a required variable but failing to operationalize it as a TDC.

h TDC: time-dependent covariate.

i Not applicable.

At prompt B, all 6 Chat and Code runs produced O1 cohorts of 5794 (−184 vs the reference 5978), attributable to a strict-inequality boundary error that was identical across all repetitions. The 2 highest-quality SAPs (B_Chat_1, B_Cowork_2) produced the worst outputs: B_Chat_1’s script entered VA categorically despite the SAP specifying continuous per–5-letter modeling and crashed; B_Cowork_2’s Python implementation included injection count in the Cox formula despite its SAP explicitly excluding it (HR 1.329 vs reference 1.434). While B_Cowork_1, with the least specific SAP, independently generated the most accurate cohort definitions in code. This trend suggests that SAP quality and implementation accuracy may represent distinct dimensions that did not covary in this small sample (Table S4 in Multimedia Appendix 1).

At prompt C, all completed runs achieved the reference cohort size. However, a consistent new error emerged: every run that adopted the counting-process structure also included the injection count in the Cox formula despite the SAP’s explicit exclusion. The SAP provided rationale for the exclusion, yet the agent reproduced only the observable structural pattern without operationalizing the stated omission. Code mode was the most reproducible; Cowork mode substituted Python for R in multiple runs, with one (C_Cowork_2) producing a concordance inflated from 0.749 to 0.867 due to interval oversplitting (83,088 rows vs the reference 35,949), which was undetectable from model output alone (Table S5 in Multimedia Appendix 1).

Post hoc diagnostic testing of the reference Cox PHs models revealed that several key variables violated the PHs assumption, and that the original R code omitted patient-level clustering in the Cox model. However, in the original 27 unguided runs, the agents blindly replicated this unclustered reference model and completely omitted PHs and multicollinearity diagnostics (only C_Code_3 noted the PH assumption in its text plan, but failed to write any code to evaluate it). In our post hoc update phase (Table S6 in Multimedia Appendix 1), when explicitly prompted to run diagnostics and resolve violations, the agents successfully executed variance inflation factor checks in 17 of 18 runs. In runs C_Code_2 and C_Code_3, the agents automatically resolved the PH violations without prompt requesting. These results illustrated that while agents lack spontaneous diagnostic metacognition, they can implement standard statistical verification and model refinement checks reliably when guided by a prescriptive protocol under human oversight.

### Script Output to Result Text Fidelity (Prompts B and C)

For most prompt B runs, numerical values in the result summaries accurately reflected the execution logs, without detectable hallucinations (Table S7 in Multimedia Appendix 1). B_Chat_2 and all B_Code runs modeled VA per individual letter; the resulting HR of 1.075 per letter was accurately transcribed but clinically nonstandard (conventional scale: HR^5≈1.44), requiring clinician manual conversion.

For prompt C, the Chat and Code run summaries accurately reflected the logs. C_Chat_1 described an O2 cohort consistent with its log, and also faithfully transcribed a code error reflecting its incorrect cohort definition, without hallucination. C_Cowork_2 reported an inverted O2 HR direction that propagated to a confident but clinically incorrect narrative interpretation (“higher baseline VA was paradoxically associated with shorter duration of sustained ≥70”), demonstrating how implementation errors can produce misleading result text that is undetectable without expert code review.

### Narrative Result Summary Quality (Prompts B and C)

Narrative summaries were of high quality in most runs (Table 2). Of 17 evaluated summaries, 8 were rated satisfactory across all 3 criteria (interpretation, clinical implications, and reporting alignment); 7 had minor issues in one dimension; and 2 had notable deficiencies across multiple criteria. Clinical implications was the most consistently handled dimension: all runs except C_Cowork_2 produced clinically sound messages covering early treatment benefit, induction adherence, and age-stratified counseling. Causal language was managed well in most runs; the best performers (B_Chat_2, B_Chat_3, C_Chat_3, and C_Code_2) explicitly disclaimed causal inference within the analysis section rather than deferring to the limitations section alone.

**Table 2.** Narrative result summary quality by run (prompts B and C)^a^.

Run	Interpretation	Clinical implications	Reporting alignment	Key finding
B_Chat_1	▲^b^	✓^c^	✓	Near-causal phrasing for drug effect (“independent treatment-level effect”); otherwise correctly associative
B_Chat_2	✓	✓	✓	Best in B-Chat group; explicit causal disclaimer; C-stat differences noted across outcomes
B_Chat_3	✓	✓	✓	Causal inference disclaimed for drug comparison; HR^d^ per letter but mathematically consistent
B_Code_1	▲	✓	▲	Reports ethnicity effect not in reference paper; limited O2 discrimination discussion
B_Code_2	✓	✓	✓	Clean associative language; drug nonsignificance for O1 and O2 correctly stated
B_Code_3	▲	✓	▲	O2 injection-interval association not in reference paper; minor O3 magnitude discrepancy
B_Cowork_1	✓	✓	✓	Confounding by indication explicitly caveated; per–5-letter HR aligned with paper
B_Cowork_2	✓	✓	▲	Methodologically sophisticated; attenuation of drug effect with injection covariate not flagged as diverging from paper
B_Cowork_3	▲	✓	✗^e^	Drug effect on O1 reported as significant and clinically conclude as superiority, whereas original paper finds no drug effect on O1
C_Chat_1	▲	✓	▲	Drug effect magnitude differs from paper; partial caveat; time-varying injection inverse correctly interpreted
C_Chat_2	✓	✓	▲	Age nonsignificant in O1, which diverges from paper (where HR 0.88/5 y)
C_Chat_3	✓	✓	✓	Best in C-Chat group; explicitly notes real-world era effects; paradoxical injection effect correctly explained
C_Code_1	✓	✓	▲	Drug nonsignificant across all outcomes, which diverges from the original paper
C_Code_2	✓	✓	✓	Exceptionally written; drug effect caveat well-framed; all 3 outcomes fully covered
C_Code_3	✓	✓	✓	Drug effect O3 correctly highlighted; proportional hazard assumption noted; induction covariate addressed
C_Cowork_1	✓	✓	✓	Confounding by indication caveated; O3 drug magnitude aligned with paper
C_Cowork_2	▲	▲	▲	O2 direction inverted (“higher baseline VA^f^ paradoxically shorter sustain”); counterintuitive direction not resolved; multiple issues across criteria
C_Cowork_3	—^g^	—	—	No output (execution failure)

a Evaluation criteria: interpretation (associative language; correct direction and magnitude); clinical implications (logical, proportionate, and caveated); and reporting alignment (key paper findings covered; no invention).

b Minor issue.

c Satisfactory.

d HR: hazard ratio.

e Notable issue.

f VA: visual acuity.

g Not applicable.

Two runs had clinically meaningful errors. B_Cowork_3 incorrectly reported a significant drug effect on O1 (time to achieving VA≥70) and derived a clinical implication of aflibercept superiority for this outcome, contradicting the reference paper’s finding. C_Cowork_2 produced a directionally inverted interpretation of the O2 finding, stating that higher baseline VA was associated with a shorter duration of sustaining at least 70 letters, which arose from the implementation bias identified at the execution stage and propagated into a confident but incorrect clinical conclusion.

## Discussion

This study evaluated an LLM agent across 3 clinical data analysis stages using a validated ophthalmology dataset and a fully specified reference analysis, in alignment with our primary objective of characterizing agent reliability, systematic failure modes, and required human oversight. The LLM agent was validated across different clinical data analysis stages, moving from a clinical question to an SAP, then to time-to-event code and narrative interpretation, which is common across observational research.

Relative to our stated aims, our main findings are as follows: for research question formulation, the agent successfully generated clinically relevant and important research questions, but their novelty was moderate, and 3 questions had low analytical feasibility due to data availability constraints (required clinical variables were absent from the raw CSV file).

The agent drafted complete, technically compliant SAPs, but we observed a critical dissociation where high-quality SAPs did not predict code correctness, and the agent introduced silent, plausible-looking execution errors (such as cohort boundary mismatches, time-zero tied survival time omissions, and incorrect censoring definitions) that produced no runtime error signals. Guided runs with detailed SAPs resolved cohort boundaries but introduced other repetitive error types, where the agent reproduced data structures from the SAP while ignoring explicit covariate exclusion directives. For transcription of numerical results from code logs to narrative summaries, the agent’s performance was highly accurate, but we identified in one case that the agent propagated an inverted HR into a confident but clinically incorrect narrative conclusion.

These findings suggest that while LLM agents can accelerate the initial generation of research questions, study protocols, and analysis code, they are currently better suited to supporting analytical work under strict expert supervision than to autonomously conducting it. Indeed, the substantial “verification time-tax” required to audit the code line-by-line, coupled with the absence of a pre-established ground truth in real-world practice, means that autonomous deployment carries significant risks of introducing silent, plausible-looking statistical errors that a researcher cannot easily identify without prior expertise.

For research question formulation, while all modes generated clinically grounded questions, mode selection shaped the thematic range of the output but did not map cleanly onto clinical value. Chat generated one treatment-burden question; Code generated trajectory phenotyping and withheld-injection clustering questions; and Cowork was the only mode to generate questions on ethnic disparity mediation. This pattern is consistent with lower-autonomy modes defaulting toward the most structurally prominent variables, though causal attribution to mode alone cannot be established from a nonrandomized design in which modes also differ in tool access, interface, and computational environment [16,17]. Despite this, the most divergent questions generated by higher-autonomy modes (such as ethnic-disparity mediation and withheld-injection clustering) had low feasibility because they required access to variables weakly represented in the released CSV (eg, socioeconomic status, comorbidity measures, and explicit noninjection visit coding). In addition, while the generated questions were highly important (mean score 4.2/5, SD 0.43) and reasonably feasible (mean score 3.5/5, SD 0.92), their novelty was moderate (mean score 3.0/5, SD 0.92) as many repeated familiar themes from the published literature. Consequently, clinician-researchers using the agent for question generation should review novelty, clinical priority, and dataset support separately rather than treating a well-formed question as study-ready.

The dissociation between SAP quality and code correctness is consistent with the current LLM architecture, in which language generation and code generation are functionally distinct processes [18,19], yet other explanations are also possible, such as context window limitations. Providing a complete, rationale-annotated SAP at prompt C resolved cohort boundary errors, as the agent operationalized explicit eligibility criteria correctly. However, this is still not a solution for all errors. Although every run reproduced the counting-process data structure described in the SAP, they included the injection count variable that the SAP explicitly excluded from the model formula. The SAP stated the exclusion and explained why, while the agent reproduced the observable structural pattern without operationalizing the stated omission. Thus, the practical lesson is that the verification should focus on the model formula itself instead of the stated SAP.

Beyond structural coding mistakes, several failure modes highlighted a lack of robust commonsense medical reasoning and metacognition. For example, B_Code iterations modeled VA down to the single ETDRS letter rather than defaulting to the standard 5-letter. Additionally, agents iteratively struggled to handle injection counts as a time-varying covariate, reproducing counting-process frameworks without conceptually integrating why treatment frequency necessitates specific statistical handling. Agents also narrated directionally inverted HR for VA endpoints, constructing plausible-sounding clinical summaries that completely failed to recognize the inherent biological paradox of their own conclusions. Furthermore, the agents failed to demonstrate basic statistical diagnostics spontaneously. In the original 27 runs, only one (C_Code_3) noted the PHs assumption, and none evaluated it or checked for multicollinearity. The agents executed the prespecified Cox models and KM curves blindly, ignoring significant violations of model assumptions (such as the PHs violation of baseline VA and the moderate multicollinearity between drug regimen and treatment era). However, in our post hoc update phase, when explicitly prompted to execute these diagnostics, the agent successfully wrote and executed PHs testing in 17 of the 18 runs. This indicates that while agents lack spontaneous diagnostic metacognition, they can only implement standard statistical verification checks reliably when guided by a prescriptive protocol under human oversight. Together, these errors underscore that while an LLM can mimic the syntax of clinical data analysis, it lacks the critical metacognition required to independently sanity-check its inputs, model design, and interpretations against real-world clinical logic, which is echoed by other studies [20,21].

A methodological concern when benchmarking LLM agents on retrospective data is data contamination. To assess the contamination status of our benchmark, we conducted an empirical zero-shot probing study by querying the LLM in independent sessions without any attached files [22]. These probes evaluated the model’s direct recall of the raw dataset schema, the original R analysis script, and the published results. The results revealed that while the model could retrieve the paper’s final text-based results (such as cohort sizes and specific HRs), it had no memory of the raw dataset schema (recalling incorrect variable names from a third-party R package) or the R script’s code syntax (such as the specific tmerge or counting-process implementation). One of the key findings is that we observed direct conflicts between the model’s zero-shot memory and its actual execution outputs: the model recalled the published cohort size of 5978 but dynamically output 5794 eyes due to strict-inequality code logic, and recalled the baseline VA HR of 1.43 but output a biased 1.329 HR due to formula composition errors. The agent’s language summaries anchored exclusively on these local execution logs rather than correcting them using pretraining memory, confirming that the agent was dynamically generating and executing code. Furthermore, to verify file-level noncontamination, we probed the model for unpublished dataset statistics (the mean and missing count of the mean_inj_interval column), which the model was unable to retrieve. Together, these findings indicate that the benchmark effectively measures the agent’s task execution capacity, even when the underlying study’s high-level findings are present in the model’s training data. We acknowledge that other contamination detection methods are available [23,24]; however, dense clinical and biostatistical text inherently resists word-level synonym swapping, as key clinical terms (drug names, ophthalmic measurement scales, defined statistical model names, and numerical outcome parameters) cannot be substituted without altering clinical meaning or creating nonsensical prose. More fundamentally, both methods measure the degree of text memorization, whereas the central validity question for our study is the knowledge-to-execution transfer: whether text-level recall translates into memorized code execution. Our memory-to-execution dissociation provides direct evidence that code execution was dynamic and situated rather than memorized. Even conservatively assuming that partial text-level contamination influenced the agent’s SAP method selection (eg, choosing Cox regression and survival analysis), the model still produced systematic coding errors across all 18 execution runs, which supports our implementation-level error findings.

Recent literature has increasingly explored the utility of LLMs in statistical analysis, reviewing their general capabilities in exploratory data analysis and code generation [25,26], or demonstrating proof-of-concept autonomous research pipelines [27,28]. While these studies acknowledge that LLMs exhibit “nonstandard errors” arising from variable analytical choices and emphasize the necessity of human copiloting for complex tasks [29], they frequently evaluate performance on isolated coding challenges [26] or general nonclinical domains [28,30]. Our study is distinguished by evaluating an LLM agent across the complete, end-to-end clinical data analysis workflow using a validated, real-world clinical dataset. Consistent with previous reports, we found that while LLMs efficiently generate plausible code and text, they lack robust statistical domain-aware error-checking, require human verification [29,31], and can produce silent execution errors [26]. However, our findings diverge by characterizing silent failure modes and a critical dissociation in which high-quality statistical analysis plans do not guarantee correct code implementation [26]. Whereas prior work often provides generic warnings regarding LLM hallucinations [27,32], our study provides actionable, targeted guidance for clinical researchers. Our findings indicate that analysis plans or numerical outputs alone cannot be assumed to confirm analytical correctness [26-28] and propose a concrete oversight framework that mandates expert verification of specific components of the code before AI-assisted research can be safely interpreted for clinical practice [29].

A further contribution of this study is methodological. Beyond evaluating one model on one dataset, we propose a reusable multistage evaluation framework for prospectively assessing future model releases. Rather than proposing our specific dataset-script replication task as a static benchmark, which would be highly vulnerable to benchmark leakage and memorization once published, this framework serves as a methodological blueprint. It translates our findings into a reusable infrastructure (the error taxonomy in Table 3 and the minimum evaluation framework in Table 4) that researchers can apply to their own local, unpublished clinical datasets. With specialty-appropriate datasets and reference analyses, the same framework could be applied to other clinical domains to support local validation before deployment. In real-world analytic tasks where no published reference analysis is available, the error taxonomy and evaluation framework may also help structure oversight by identifying high-risk stages of the workflow, prioritizing targeted verification checks, and documenting where errors arise, although they do not replace expert validation.

**Table 3.** Error pattern taxonomy: observed large language model (LLM) agent failure modes by evaluation stage.

Error pattern	Example	Likely mechanism	Potential mitigation
SAP^a^ errors			
Within-mode SAP stereotyping	All 3 B_Code SAPs: identical gaps in VA^b^ scaling and injection count handling	Agent converges on a fixed SAP template within a mode	Cross-mode SAP comparison; use Chat mode when precision is required
Blurry description	B_Chat_2: “injection count may be used as a covariate”	Permissive language interpreted as nonobligatory	Use prescriptive directives; avoid “may,” “consider,” “if appropriate”
SAP-to-analysis execution errors			
SAP-script dissociation	B_Chat_1: SAP specifies VA per 5 letters; script enters VA categorically and crashes	SAP and code generation are functionally distinct LLM processes	Expert comparison of model formula against SAP; treat as separate steps
Cohort boundary error	All B_Chat and B_Code runs: O1 n=5794 (−184) due to strict vs nonstrict inequality	Default to strict inequality without explicit boundary specification	Specify eligibility criteria with explicit boundary condition
Language substitution artifact	C_Cowork_2: Python substituted for R; concordance 0.749 → 0.867 due to interval over-splitting	Agent selected Python; library-specific differences introduced bias	Specify programming language; check counting-process row count in log
Lack of basic clinical judgment	B_Chat_2, B_Code_1‐3: treating VA as per 1 letter or categorical variable. All prompt C runs: counting-process structure adopted but injection count included in Cox formula despite SAP exclusion	LLMs reproduce observable positive patterns more reliably than deliberate omissions	Annotate SAP exclusions with rationale; verify model formula line-by-line
Lack of self-validation and refinement	B_Code runs: failed to spontaneously check model assumptions (proportional hazards violations) or run VIF^c^ diagnostics, and seldomly refined the statistical model until prescriptively prompted	Agent prioritizes completion of prespecified script execution without statistical self-validation or metacognition	Mandate model diagnostics (Schoenfeld residuals, VIF) and model refinement rules directly in the SAP
Script-to-text errors			
Postcrash rerun undisclosed	B_Chat_1: summary reflects corrected rerun, not the crashing script	Agent generates summary after resolving error; does not disclose substitution	Verify summary corresponds to the same analysis.log as the reported script
Biased result → misleading narrative	C_Cowork_2: inverted O2 HR^d^ → clinically incorrect but confident interpretation	Biased result interpreted without reference to expected association direction	Expert review of HR direction and concordance before reporting
Script-to-narrative errors			
Spurious clinical claim	B_Cowork_3: reports significant drug effect on O1 and concludes drug superiority	Agent hallucinated significance or overinterpreted finding, contradicting reference paper	Expert clinician review of narrative conclusions against raw statistical outputs
Paradoxical rationalization	C_Cowork_2: confidently explains inverted relationship between baseline VA and sustained duration	Agent constructs plausible narrative to fit biased execution output, failing to recognize the clinical paradox	Mandate a “clinical sanity check” to evaluate biological plausibility of results

a SAP: statistical analysis plan.

b VA: visual acuity.

c VIF: variance inflation factor.

d HR: hazard ratio.

**Table 4.** Minimum evaluation framework for large language model (LLM) agents in clinical data analysis.

Framework element	What it is	How to use it for a new agent or model release	Example from this study
1. Reference-anchored benchmark	Evaluate the agent against a real dataset with a validated reference analysis, not just subjective impressions.	Choose a specialty dataset with a known analysis pipeline and prespecified reference outputs.	The study used a public ophthalmology dataset plus a validated reference script and output as the ground truth comparator.
2. Stage-based pipeline evaluation	Separate the workflow into distinct stages rather than scoring only the final answer.	Keep the stages separate in future evaluations so strengths in one stage do not hide failures in another.	The study evaluated question generation, SAP^a^ drafting, execution, result text, and narrative summary as different constructs. Strong SAPs did not guarantee correct analytic code implementation.
3. Graduated prompt levels	Test different degrees of task and aim specification.	Reuse the same 3 levels to test whether a model improves when guidance increases.	The framework used prompt A (data to questions), prompt B (aim to SAP plus execution), and prompt C (SAP to execution). Prompt C reduced cohort-boundary errors but did not eliminate formula errors.
4. Structural code review, not output review alone	Inspect formulas, eligibility rules, and data structure, not just headline estimates.	For future releases, require inspection of formula composition, cohort logic, and row structure before accepting outputs. Ask the agent to do diagnosis analysis or refinement of the modeling.	Injection count was wrongly included in Cox models despite SAP exclusion; Python interval over-splitting inflated concordance; model conducted prespecified SAP blindly though it has the capability to diagnosis the issues of the current modeling and conduct refinement.
5. SAP-to-code fidelity check	Treat planning quality and coding quality as distinct dimensions.	Always compare the code directly against the SAP instead of assuming faithful execution.	One of the best SAPs produced one of the worst implementations.
6. Clinician interpretation review	Evaluate whether the narrative is clinically logical, not just statistically grammatical.	Add expert review for effect direction, causal language, and biological plausibility.	Visual acuity was modeled down to the single ETDRS^b^ letter rather than defaulting to the standard 5-letter. Inverted hazard-ratio direction was turned into a confident but clinically incorrect explanation.
7. Error taxonomy linked to audit steps	Convert recurring failures into a reusable checklist.	Reuse the taxonomy prospectively when testing new releases; track whether old failure classes disappear and new ones emerge.	The study’s Table 3 classifies failures by mechanism and mitigation.

a SAP: statistical analysis plan.

b ETDRS: Early Treatment Diabetic Retinopathy Study.

Our study has limitations. First, this is a single-dataset, single-agent pilot study. Second, the dataset used in this study has been manually cleaned so the findings may only be representative of performance on precleaned datasets, with qualitative evaluation anchored by comparison with a validated reference. Further multiagent, multidataset benchmarking is required to generalize across platforms or analytical domains. Moreover, our zero-shot probing showed the model could retrieve the published text-level results of the reference study, which may be aided by its use of live web-search tools. While we cannot definitively establish whether the original paper itself was present in the pretraining corpus, we acknowledge that this may have influenced its selection of survival analysis methods and covariates during SAP generation. Although our memory-to-execution dissociation analysis provides direct evidence that code execution was dynamic rather than memorized, we cannot fully exclude the possibility that the agent’s high-level analytical design was shaped by pretraining knowledge of the published methodology. Future studies should evaluate LLM agents on unpublished clinical datasets using our proposed evaluation framework (Table 4) and error taxonomy (Table 3) as a reusable blueprint to assess performance under conditions free of any potential text-level contamination.

In conclusion, LLM agents are best regarded as capable analytical assistants requiring a mandatory expert-verification layer rather than autonomous biostatisticians. While they can dramatically accelerate the initial draft stages of research, spanning writing protocols, generating questions, and drafting template code, their code execution remains prone to introducing silent, plausible-looking errors that can compromise clinical validity. Therefore, even when used as analytical support tools, their outputs require critical validation.

The broader implication is that safe adoption will depend not only on better models but also on rigorous, reusable evaluation frameworks. The utility of generative AI in clinical research is highly asymmetrical, benefiting experienced clinical data scientists who can quickly audit code but carrying high risks for novice researchers who cannot verify the underlying code or statistical logic. The “verification time-tax” required to check the implementation status of the SAP by the model significantly offsets any automation speedups, making autonomous AI deployment in peer-reviewed clinical research premature. Evaluating AI models based on execution success (ie, whether the code runs without throwing errors) is insufficient for safety-critical clinical informatics. Benchmarks must shift toward auditing logical correctness. This study yields a stage-based minimum evaluation framework for future LLM agents in clinical data analysis (Table 4). Although demonstrated in an ophthalmology dataset, the workflow and oversight logic are adaptable across clinical specialties.

## Supplementary material

10.2196/99597 Multimedia Appendix 1 Observed capabilities, limitations, and responsible use of a large language model agent for clinical data analysis.

## Abbreviations

DECIDE-AI: Developmental and Exploratory Clinical Investigations of Decision Support Systems Driven by AI
ETDRS: Early Treatment Diabetic Retinopathy Study
HR: hazard ratio
HRA: Health Research Authority
JAMA: Journal of the American Medical Association
KM: Kaplan-Meier
LLM: large language model
NHS: National Health Service
PH: proportional hazard
SAP: statistical analysis plan
VA: visual acuity
