# Supplementary material for: Performance, Failures, and Oversight of a Large Language Model Agent for Clinical Data Analysis: Evaluation Study
Source: J Med Internet Res. 2026 Sep 8;28:e99597. doi: 10.2196/99597 (PMC13552643; doi:10.2196/99597)
Supplement: Supplementary file 1 [file jmir-v28-e99597-s001.docx]

# **Appendix to:**

# **Large Language Model Agent for Clinical Data Analysis: Observed Capabilities, Limitations, and a Framework for Responsible Use**

## Appendix Contents

- Method S1 — Detailed Prompt Specifications
- Method S2 — Study Design Overview: Pipeline Stages, Prompt Levels, and Evaluation Framework
- Method S3 — Statistical Analysis Plan (SAP) Evaluation Reference Checklist
- Method S4 — Reference Analysis Script Key Features
- Method S5 — Zero-shot memory probing to test model contamination
- Table S1 — Research Questions Generated by the AI Agent (Prompt A, 18 Questions Across 9 Runs)
- Table S2 — SAP Quality by Run (Prompt B, 9 Runs)
- Table S3 — Appendix Analytical Methods Proposed by the Agentic SAPs (Prompt B, 9 Runs)
- Table S4 — Structural Feature Comparison: Reference Script vs Agent Implementations
- Table S5 — Full Quantitative Results: Cohort Sizes, KM Medians, Hazard Ratios, and Concordance (All 18 Prompt B, C Runs vs Reference)
- Table S6 — Proportional Hazard Diagnostics Comparison (Prompt B and C, 18 Runs)
- Table S7 — Script Output to Result Text Fidelity by Run
- Table S8 — DECIDE-AI Reporting Guideline Checklist

## Method S1 — Detailed Prompt Specifications

All prompts were delivered within the Claude desktop interface. Each run operated in an entirely independent session (no shared context, memory, or files between runs). The prompt text was identical for all mode–Prompt Combinations within the same level; the only variation was the selection of agent interaction mode (Chat, Code, or Cowork) in the platform interface.

### A Prompt

*"This is a longitudinal dataset of visual outcomes from eyes receiving anti-VEGF treatment for age-related macular degeneration. Read Original/data_dictionary.rtf and Original/dataset.csv. Write exactly TWO research questions that you think are novel, important, and feasible, and save to raw/llm_outputs/questions.md."*

The agent received access to the data dictionary and dataset only. No research objective, outcome definitions, or any prior analytical context was provided.

### B Prompt

*"This is a longitudinal dataset of visual outcomes from eyes receiving anti-VEGF treatment for age-related macular degeneration. Read Original/data_dictionary.rtf and Original/dataset.csv. We want to conduct survival analysis for the three clinical outcomes including: 1 — VA reaching ETDRS score 70 or better; 2 — duration of VA sustained at or better than 70; 3 — VA declining to 35 or worse. Please provide a statistical analysis plan that should include: [1] statistical analysis models; [2] data inclusion and exclusion criteria; [3] variables included in the analysis models. Write the SAP and save to raw/llm_outputs/sap.md."*

Following SAP generation, the agent was then asked to: implement the SAP in R (or equivalent); execute it on the dataset; save metrics, cohort flow, and session information; and write results_summary.md and conclusion_summary.md summarizing the outputs:

“*Write raw/llm_outputs/analysis.R implementing the SAP to the dataset exactly. Run it; save metrics.json, cohort_flow.json, sessionInfo.txt, logs in raw/r_outputs. Write results_summary.md and conclusion_summary.md using the above computed outputs. The results_summary.md should directly report the key statistical analysis results, including but not limited to the sample size, baseline descriptions, method, variables and results for each analysis conducted. The conclusion_summary.md should be high-Prompt Conclusion in a manuscript-like style narrative.*”

### C Prompt

*"This is a longitudinal dataset of visual outcomes from eyes receiving anti-VEGF treatment for age-related macular degeneration. Read Original/data_dictionary.rtf and Original/dataset.csv. We want to conduct survival analysis for the three clinical outcomes including: 1 — VA reaching ETDRS score 70 or better; 2 — duration of VA sustained at or better than 70; 3 — VA declining to 35 or worse.*

*Our data analysis plan is: [1] For the statistical analysis model, use Cox proportional hazards model and Kaplan-Meier estimator for the three clinical outcomes. [2] For the variables included in the analysis: For Cox proportional hazards regression model: covariates include sex, ethnicity, age at baseline, VA at baseline, drug type, treatment start date, number of anti-VEGF injections, induction phase completed, injection interval. For Kaplan-Meier estimator: subgroup for age at baseline, VA at baseline, drug type. Mind that it is common sense that VA is scaled by 5 ETDRS letters and injection number is a time-dependent covariate. [3] For the reporting items of each outcome, include at least sample size of each included cohort, HR for baseline VA, and median time in years.*

*Write raw/llm_outputs/analysis.R implementing the SAP to the dataset exactly. Run it; save metrics.json, cohort_flow.json, sessionInfo.txt, logs in raw/r_outputs. Write results_summary.md and conclusion_summary.md using the above computed outputs. The results_summary.md should directly report the key statistical analysis results, including but not limited to the sample size, baseline descriptions, method, variables and results for each analysis conducted. The conclusion_summary.md should be high-Prompt Conclusion in a manuscript-like style narrative."*

## Method S2 — Study Design Overview: Pipeline Stages, Prompt Levels, and Evaluation Framework

| **Pipeline Stage** | **Prompt** | **Agent Input** | **Agent Output** | **Evaluation Method** | **Key Construct Assessed** |
| --- | --- | --- | --- | --- | --- |
| **Research Question Generation** | A | Dataset + data dictionary | 2 research questions | Qualitative thematic grouping (7 domains, derived inductively); mode convergence examined | Thematic diversity; mode-dependent divergence |
| **SAP Drafting** | B | Outcome definitions (3 survival endpoints) | Complete statistical analysis plan | 10-item reference checklist; descriptive characterisation per dimension | VA scaling, TDC specification, injection count exclusion, covariate coverage |
| **Analysis Execution** | B | Outcome definitions + agent-drafted SAP | Analysis script + execution logs | Quantitative output comparison vs reference (cohort n, KM median, HR per 5 VA letters, concordance) + structural code review | Cox formula accuracy; cohort boundary logic; counting-process implementation |
| **Analysis Execution** | C | Investigator-drafted SAP (fully specified) | Analysis script + execution logs | Same as Prompt B | Same as Prompt B; additional assessment of SAP-to-code fidelity |
| **Script → Result Text** | B, C | Execution log outputs | Numerical results summary | Line-by-line fidelity check: summary vs execution log; numerical accuracy and disclosure audit | Transcription accuracy; unit propagation; undisclosed deviations |
| **Script → Manuscript** | B, C | Execution log outputs | Clinical narrative with interpretation | Clinician review on 3 criteria: (A) Interpretation, (B) Clinical Implications, (C) Reporting Alignment | Causal language calibration; clinical implication accuracy; paper alignment |

27 total runs: 9 Prompt A (3 modes × 3 repetitions) + 9 Prompt B (3 modes × 3 repetitions) + 9 Prompt C (3 modes × 3 repetitions). All runs conducted in fully independent sessions with no shared context or memory.

Prompt A produces questions only; Prompt B produces SAP + analysis + summaries; Prompt C produces analysis + summaries (no SAP).

Cowork mode runs at Prompts B, C used Python (lifelines) for runs 1–2; all other runs used R.
Abbreviations: KM, Kaplan-Meier; SAP, statistical analysis plan; TDC, time-dependent covariate; VA, visual acuity.

## Method S3 — SAP Evaluation Reference Checklist

### Clinical Outcome Definitions (Reference Standard)

| **Outcome** | **Label** | **Cohort Eligible** | **Event** | **Censoring** |
| --- | --- | --- | --- | --- |
| O1 | Time to VA ≥70 ETDRS letters | Eyes with baseline VA <70 (n = 5,978) | First visit with VA ≥70 | End of follow-up without achieving VA ≥70 |
| O2 | Duration of sustained VA ≥70 letters | Eyes that ever achieved VA ≥70 (n = 4,439) | First visit where VA dropped below 70 after achieving ≥70 | End of follow-up with VA maintained ≥70 |
| O3 | Time to VA ≤35 ETDRS letters | Eyes with baseline VA >35 (n = 6,453) | First visit with VA ≤35 | End of follow-up without declining to VA ≤35 |

Each Prompt B SAP was evaluated descriptively by a clinician-researcher reviewer against a reference checklist of 10 analytical dimensions derived from the reference analysis. For each dimension, the SAP was characterised as fully specified, partially specified or ambiguous, or absent, with the key language from the SAP noted where relevant. The dimension-by-dimension characterisation for each run is reported in Table S5.

| **Item** | **Criterion** | **Rationale** |
| --- | --- | --- |
| 1 | VA modeled as a continuous variable, scaled per 5 ETDRS letters | Per-letter parameterization yields HRs that are numerically accurate but clinically uninterpretable without conversion |
| 2 | Injection count specified as a time-dependent covariate (TDC) | Injection count changes with each visit; treating it as a baseline covariate introduces immortal time bias |
| 3 | Injection count explicitly excluded from Cox model formula | The reference model uses the TDC structure for interval construction only; the marginal effect of injection count is not the analytical target and its inclusion biases other HRs |
| 4 | O1 cohort defined as eyes with baseline VA <70 (strict inequality) | Eyes with exactly VA=70 at baseline cannot achieve the O1 outcome |
| 5 | O2 cohort defined as eyes that ever achieved VA ≥70 | O2 has a distinct cohort including eyes achieving the threshold at baseline |
| 6 | O3 cohort defined as eyes with baseline VA >35 (strict inequality) | Eyes at exactly VA=35 at baseline cannot decline to the O3 threshold |
| 7 | All 9 specified covariates included in Cox model | Missing covariates reduce comparability with the reference |
| 8 | Kaplan-Meier estimator specified for all three outcomes | Reference uses KM for non-parametric survival estimation |
| 9 | Cox proportional hazards model specified for all three outcomes | Reference uses Cox PH as the primary inferential model |
| 10 | Reporting items specified: cohort size, KM median survival, HR for VA per 5 letters | Ensures outputs are comparable to the reference and interpretable for clinical readers |

## Method S4 — Reference Analysis Script Key Features

The validated reference R script (reference_script_original.R) used the following key implementation decisions, which served as the evaluation standard for agent-generated scripts:

| **Feature** | **Reference Implementation** | **Clinical/Statistical Rationale** |
| --- | --- | --- |
| **Time-varying covariate structure** | tmerge() counting-process framework (survival package) | Correctly handles injection count as a time-dependent covariate that changes with each injection visit |
| **Cox formula** | va_inj1 + age_group + gender + ethnicity + date_inj1 + loaded + mean_inj_interval + regimen (8 terms; injnum excluded from formula) | injnum is used to construct the counting-process intervals but its marginal effect on the outcome is not the analytical target |
| **VA scaling** | va_inj1 entered as a continuous variable per ETDRS letter; HR reported per 5 letters as exp(5 × coef) | Per-5-letter scaling aligns with the minimum clinically important difference for ETDRS VA |
| **O2 KM structure** | Simple per-patient Surv(tto70/365, status) — NOT the counting-process structure | O2 event time is per-patient (time to loss of VA ≥70 from achievement), not visit-level |
| **Cohort boundaries** | Strict inequality: va_inj1 < 70 for O1; va_inj1 > 35 for O3 | Eyes at exactly the threshold value are neither in the risk set nor can achieve the outcome |
| **Reference row counts** | O1 Cox: 35,949; O2 Cox: 23,944; O3 Cox: 62,086 | Counting-process rows derived from injection-visit splitting only; all-visit splitting inflates counts |
| **Programming language** | R (survival, survminer, tidyverse packages) | — |

## Method S5 — Zero-shot memory probing to test model contamination

- Probe 1: Direct Schema Recall (Dataset Column Names)

Prompt: I am studying clinical informatics. There is a paper published in JAMA Ophthalmology in 2021 titled "Insights from survival analyses during 12 years of anti-VEGF therapy for neovascular AMD" by Fu et al. This study released its dataset publicly. Can you tell me what the specific columns (variables) of this dataset are, and their exact names?

LLM Response: “Dataset: amd (eyedata R package, 6,696 rows × 23 variables... Column names: patID, sex, age, avdays_induc, ethnicity, loaded, time, injgiven, va, regimen, pre2013... This is the curated R-package version, not necessarily the raw CSV column names. The original Dryad deposit (MEH_AMD_survivaloutcomes_database.csv) ships with its own README_for_Moorfields_AMD_Database.rtf, which I could not access — Dryad's robots.txt blocks automated retrieval. You'd need to download that file yourself directly from the Dryad page to confirm the raw CSV's exact column headers...”

- Probe 2: Reference Script Logic Recall (R Code)

Prompt: For the paper published in JAMA Ophthalmology in 2021 titled "Insights from survival analyses during 12 years of anti-VEGF therapy for neovascular AMD" by Fu et al., they released an R script to run Cox proportional hazards models and Kaplan-Meier analyses. Do you know how they set up the time-dependent covariate for injection count in that R script? Can you write out the exact R code block or describe the data manipulation steps (such as using tmerge or counting process formats) they used to link clinic visits, visual acuity, and injections?

LLM Response: "No mirror exists on GitHub. The R script is exclusively on Dryad, and Dryad's robots.txt blocks the file fetch — I can't pull the actual code. I won't reconstruct or guess at the exact script, since that would risk presenting fabricated code as if it were the real thing... To get the real code, I'd suggest downloading directly yourself..."

- Probe 3: Published Results Recall (Text-Level Memorization)

Prompt: In the paper published in JAMA Ophthalmology in 2021 titled "Insights from survival analyses during 12 years of anti-VEGF therapy for neovascular AMD" by Fu et al., what are the exact cohort sizes (N), median survival times (in years), and hazard ratios (HR) reported for the three outcomes: (1) achieving VA >= 70, (2) duration of sustained VA >= 70, and (3) time to VA decline <= 35? If you don't know the exact numbers, please state that.

LLM Response: "Here are the exact figures from the paper: Overall cohort: N = 7802 eyes/patients... Outcome (1) Achieving VA ≥ 70: Denominator: 5978 of 7802 eyes (76.6%)... Median time: 2.0 years (95% CI, 1.8–2.3)... baseline VA HR 1.43 per 5-letter increment... Outcome (2) Duration of sustained VA ≥ 70: Denominator: 4439/7802... Median time sustained: 1.1 years (95% CI, 1.1–1.2)... Outcome (3) Time to VA decline ≤ 35: Denominator: 6453 of 7802... Median time: 8.7 years (95% CI, 6.6–10.8)..."

- Prompt 4: Statistical Signature Probe (Dataset Non-Leakage)

Prompt: What is the exact column mean and the number of missing (null) values of the column mean_inj_interval in the raw Dryad dataset dataset.csv from paper published in JAMA Ophthalmology in 2021 titled "Insights from survival analyses during 12 years of anti-VEGF therapy for neovascular AMD" by Fu et al.?

LLM Response: "That direct download path is disallowed by robots.txt. I'm unable to retrieve the actual MEH_AMD_survivaloutcomes_database.csv file through any tool I have access to — datadryad.org isn't in my sandboxed network allowlist (so I can't curl it directly), and web_fetch is blocked by Dryad's robots.txt for the direct file-download path... I have no way to verify whether a column called mean_inj_interval even exists in that file, let alone compute its mean or null count, without opening the actual CSV."

**Model Contamination Zero-Shot Memory Probing vs. Experimental Run Outputs**

| **Probing Dimension** | **Zero-Shot LLM Memory Response** | **Correct Reference Value (Ground Truth)** | **False LLM Execution Outputs in Runs (Run ID)** |
| --- | --- | --- | --- |
| **Dataset Schema (Column Names)** | Recalled columns of curated R package eyedata (e.g. pre2013). Stated it could not access raw Dryad CSV headers due to robots.txt. | Dryad CSV headers: patid, va_inj1, date_inj1, loaded, mean_inj_interval. | Code failed to find columns or misidentified variables due to factor levels (e.g., C_Code_3). |
| **Reference R Script Code** | Refused to write R code; noted Dryad file access is blocked by robots.txt and won't fabricate code. | Custom tmerge split, +1 day offset, clustered Cox model coxph(..., cluster(patID)). | Unguided runs omitted +1 offset, omitted clustering, and had incorrect censoring logic (e.g., B_Code_1, B_Code_2, B_Code_3). |
| **Published Results (Text Memory)** | Recalled published text values: N = 7,802; O1 cohort = 5,978; O1 median = 2.0 years; baseline VA HR = 1.43; O2 median = 1.1 years. | N = 7,802; O1 cohort = 5,978; O1 median = 2.04 years; baseline VA HR = 1.43; O2 median = 1.13 years. | **Cohort Size**: 5,794 eyes (B_Code_1, B_Code_2).  **O1 Median**: 2.40 years (`B_Code_1`), 2.38 years (`B_Chat_1`).  **O1 HR**: 1.329 (`B_Code_1`), 1.075 (`B_Chat_1`).  **O2 Median**: "Not Reached" (`B_Code_2`). |
| **Statistical Signature (Dataset Non-Leakage)** | Refused to estimate; stated it cannot access raw dataset without curl or opening the CSV. | True mean of mean_inj_interval = 51.4016 days, null count = 1,807. | Agent had to calculate from local CSV. In successful runs (e.g., B_Code_1), it calculated the true values, proving data was read locally. |

## Table S1 — Research Questions Generated by the AI Agent (Prompt A, 18 Questions Across 9 Runs)

| **Run** | **Question (abbreviated)** | **Thematic Group** | **Novelty** | **Importance** | **Feasibility** |
| --- | --- | --- | --- | --- | --- |
| A_Chat_1 Q1 | Does induction phase completion differentially modify VA trajectories across age groups? | a — Induction completeness | 3 | 4 | 5 |
| A_Chat_1 Q2 | Is there a dose-response between mean injection interval and long-term VA gain, differing by drug type? | b — Injection interval | 3 | 4 | 4 |
| A_Chat_2 Q1 | Does early treatment response (first 3 injections) predict long-term VA trajectory at 2 and 5 years? | c — Early VA response | 2 | 5 | 4 |
| A_Chat_2 Q2 | Does injection interval variability — rather than cumulative count — explain inter-patient outcome differences? | b — Injection interval | 4 | 4 | 3 |
| A_Chat_3 Q1 | Does 12-month BCVA trajectory predict long-term (≥5-year) visual outcome, varying by injection regimen? | c — Early VA response | 3 | 4 | 3 |
| A_Chat_3 Q2 | What injection burden is required to maintain vision within 5 letters of peak, and what predicts a high-burden phenotype? | d — Treatment burden | 4 | 5 | 3 |
| A_Code_1 Q1 | Does induction loading completeness independently predict long-term VA, modified by baseline VA severity? | a — Induction completeness | 2 | 4 | 5 |
| A_Code_1 Q2 | Is there a non-linear optimal injection frequency in induction with diminishing returns by drug type? | b — Injection interval | 3 | 4 | 3 |
| A_Code_2 Q1 | Does mean injection interval (continuous) predict long-term VA, with a clinically meaningful threshold modified by regimen? | b — Injection interval | 2 | 4 | 4 |
| A_Code_2 Q2 | Can LCGA identify distinct VA trajectory phenotypes differing in injection burden and baseline characteristics? | e — Trajectory phenotyping | 4 | 4 | 3 |
| A_Code_3 Q1 | Does VA response to the first three loading injections predict ≥3-year outcomes, modified by baseline VA severity? | c — Early VA response | 2 | 5 | 4 |
| A_Code_3 Q2 | Do withheld-injection visits cluster non-randomly, and is this associated with accelerated VA decline? | f — Withheld injections | 4 | 5 | 2 |
| A_Cowork_1 Q1 | Does induction loading completion modify VA trajectories differentially by drug type and baseline VA? | a — Induction completeness | 3 | 4 | 4 |
| A_Cowork_1 Q2 | Are there ethnic disparities in VA outcomes, explained by differences in induction adherence or injection interval? | g — Ethnic disparities | 3 | 4 | 2 |
| A_Cowork_2 Q1 | Does loading completion modify the relationship between injection interval and long-term VA, varying by baseline VA? | a — Induction completeness | 3 | 4 | 4 |
| A_Cowork_2 Q2 | Are ethnic disparities mediated by differential treatment adherence and loading phase completion? | g — Ethnic disparities | 3 | 4 | 2 |
| A_Cowork_3 Q1 | Does inadequate induction loading modify the baseline VA–VA trajectory association, differing by drug type? | a — Induction completeness | 3 | 4 | 4 |
| A_Cowork_3 Q2 | Does mean induction injection interval predict subsequent cumulative injection frequency, especially in older patients? | b — Injection interval | 3 | 4 | 4 |

**Summary by Mode**

| **Mode** | **Thematic groups reached** | **Groups unique to this mode** | **Novelty (Mean [Range])** | **Importance (Mean [Range])** | **Feasibility (Mean [Range])** |
| --- | --- | --- | --- | --- | --- |
| Chat (6 questions) | a, b, c, d | d | 3.17 (2-4) | 4.50 (4-5) | 3.67 (3-5) |
| Code (6 questions) | a, b, c, e, f | e, f | 2.83 (2-4) | 4.33 (4-5) | 3.50 (2-5) |
| Cowork (6 questions) | a, b, g | g | 3.00 (3-3) | 3.83 (3-4) | 3.33 (2-4) |
| All modes (18 questions) | 7 groups |  | 3.00 (2-4) | 3.22 (3-5) | 3.50 (2-5) |

**Summary by Question Group**

| **Thematic Group** | **N** | **Run IDs** | **Novelty (Mean [Range])** | **Importance (Mean [Range])** | **Feasibility (Mean [Range])** |
| --- | --- | --- | --- | --- | --- |
| a- Induction completeness | 5 | A_Chat_1 Q1, A_Code_1 Q1, A_Cowork_1 Q1, A_Cowork_2 Q1, A_Cowork_3 Q1 | 2.80 [2–3] | 4.00 [4–4] | 4.40 [4–5] |
| b- Injection interval | 5 | A_Chat_1 Q2, A_Chat_2 Q2, A_Code_1 Q2, A_Code_2 Q1, A_Cowork_3 Q2 | 3.00 [2–4] | 4.00 [3–5] | 3.60 [3–4] |
| c- Early VA response | 3 | A_Chat_2 Q1, A_Chat_3 Q1, A_Code_3 Q1 | 2.33 [2–3] | 4.67 [4–5] | 3.67 [3–4] |
| d- Treatment burden | 1 | A_Chat_3 Q2 | 4.00 | 5.00 | 3.00 |
| e- Trajectory phenotyping | 1 | A_Code_2 Q2 | 4.00 | 4.00 | 3.00 |
| f- Withheld injections | 1 | A_Code_3 Q2 | 4.00 | 5.00 | 2.00 |
| g- Ethnic disparities | 2 | A_Cowork_1 Q2, A_Cowork_2 Q2 | 3.00 [3–3] | 4.00 [4–4] | 2.00 [2–2] |

Abbreviations: BCVA, best-corrected visual acuity; ETDRS, Early Treatment Diabetic Retinopathy Study; LCGA, latent class growth analysis; VA, visual acuity.

## Table S2 — SAP Quality by Run (Prompt B, 9 Runs)

| **Run** | **VA Scaling (Item 1)** | **TDC Structure (Item 2)** | **Injection Count Excluded from Cox Formula (Item 3)** | **Cohort Criteria (Items 4–6)** | **All 9 Covariates (Item 7)** | **Reporting Items (Item 10)** | **Overall Characterisation** |
| --- | --- | --- | --- | --- | --- | --- | --- |
| B_Chat_1 | ✓ per 5 letters | ✓ TDC structure specified | ✓ Explicit exclusion with rationale | ✓ | ✓ | ✓ | Highest technical precision; only SAP to explicitly exclude injection count with stated rationale |
| B_Chat_2 | ✓ per 5 letters | ⚠ "may be used as a covariate" — permissive, non-obligatory language | ⚠ Not stated; follows from ambiguous Item 2 | ✓ | ✓ | ⚠ HR per 5 letters not required | Hedged directive: permissive language leaves injection count handling to agent discretion |
| B_Chat_3 | ✓ per 5 letters | ⚠ TDC specified for O2 only; O1 and O3 not addressed | ⚠ Exclusion stated for O2 only | ✓ | ✓ | ⚠ HR per 5 letters not required | Partial TDC coverage; incomplete across outcomes |
| B_Code_1 | ⚠ "continuous" — per-letter scaling implied, not stated | ✓ | ✓ | ✓ | ✓ | ✓ | VA unit gap; otherwise complete; all 3 Code SAPs nearly identical |
| B_Code_2 | ✗ Not mentioned | ⚠ Listed as covariate; TDC structure not named | ✗ No exclusion instruction | ✓ | ⚠ VA scaling absent; 8 of 9 effective | ⚠ Generic reporting; HR per 5 letters not required | Within-mode stereotyping; identical gaps to B_Code_3 |
| B_Code_3 | ✗ Not mentioned | ⚠ Same as B_Code_2 | ✗ No exclusion instruction | ✓ | ⚠ Same as B_Code_2 | ⚠ Same as B_Code_2 | Near-identical to B_Code_2; within-mode stereotyping confirmed |
| B_Cowork_1 | ✗ Absent | ✗ Not mentioned | ✗ Absent | ✓ | ✗ Injection count, interval, and VA scaling absent; 6 of 9 effective | ✓ | Lowest SAP specificity; yet produced most accurate cohort definitions in code |
| B_Cowork_2 | ✓ per 5 letters | ✓ tmerge structure named explicitly | ✓ Explicit exclusion with rationale | ✓ | ✓ | ✓ | Highest overall SAP quality; most technically detailed |
| B_Cowork_3 | ✗ Absent | ⚠ Time-varying noted; tmerge not named | ✗ No exclusion instruction | ✓ | ⚠ VA scaling absent; injection count ambiguous | ⚠ Reporting metrics not fully specified | Moderate SAP; consistent with mode pattern |

For each dimension, characterisation reflects the reading of the SAP text as documented in the run's output file. Abbreviations: ✓ = fully specified; ⚠ = partially specified or ambiguous (key language noted); ✗ = absent.

## Table S3 — Appendix Analytical Methods Proposed by the Agentic SAPs (Prompt B, 9 Runs)

| **Method** | **Category** | **Outcome** | **Chat_1** | **Chat_2** | **Chat_3** | **Code_1** | **Code_2** | **Code_3** | **Cowork_1** | **Cowork_2** | **Cowork_3** |
| --- | --- | --- | --- | --- | --- | --- | --- | --- | --- | --- | --- |
| Fine-Gray competing risks | Competing events | O3 (all); O1 (some) | ✓ | ✓ | ✓ | ✓ | ✓ | ✓ | ✓ | ✓ | ✓ |
| Landmark analysis | Bias correction | O2 | ✓ | ✓ | ✓ | ✓ | ✓ | ✓ | ✓ | ✓ | ✓ |
| RMST | Summary measure | O2, O3 | — | — | — | — | ✓ | ✓ | ✓ | ✓ | ✓ |
| AFT (Weibull / log-normal) | Parametric | All | — | — | — | — | ✓ | — | ✓ | ✓ | ✓ |
| Multi-state model (3-state) | Joint modelling | O1+O2 | — | ✓ | ✓ | — | — | — | ✓ | ✓ | — |
| Shared frailty Cox (gamma) | Correlated data | All | — | — | ✓ | ✓* | — | — | — | ✓ | ✓ |
| Robust sandwich variance | Correlated data | All | ✓ | — | — | ✓* | ✓ | ✓ | — | — | — |
| Recurrent events (A-G / WLW) | Repeated outcomes | O2 | ✓ | ✓ | — | — | — | — | — | — | — |
| Interval-censored Cox (icenReg) | Censoring type | All | ✓ | — | — | — | — | — | — | — | — |
| IPCW | Informative censoring | All | ✓ | ✓ | — | — | — | — | — | — | — |
| Restricted cubic splines | Functional form | Cont. covariates | ✓ | ✓ | — | ✓ | — | — | ✓ | ✓ | — |
| Time-varying Cox (injnum) | TVC | O1–O3 | ✓ | ✓ | — | — | ✓† | ✓† | ✓ | — | — |
| MICE imputation | Missing data | Covariates | ✓ | ✓ | — | ✓ | ✓ | ✓ | ✓ | — | — |
| Prediction model + bootstrap | Prognostic | O3 | ✓ | — | — | — | — | — | — | — | — |

✓* = proposed as alternative to the other; †injnum in counting-process structure but excluded from Cox formula

## Table S4 — Structural Feature Comparison: Reference Script vs Agent Implementations

| **Structural Feature** | **Reference** | **B_Chat_1/2/3** | **B_Code_1/2/3** | **B_Cowork_1** | **B_Cowork_2** | **B_Cowork_3** | **C_Chat_1/2/3** | **C_Code_1/2/3** | **C_Cowork_1** | **C_Cowork_2** |
| --- | --- | --- | --- | --- | --- | --- | --- | --- | --- | --- |
| **Counting-process structure** | tmerge | None (simple Surv) | None (simple Surv) | None (lifelines KM only) | Custom Python CP | None (simple Surv) | tmerge (2/3); custom CP (Chat_1) | tmerge (all 3) | tmerge | Custom Python CP |
| **Injection count in Cox formula** | ✗ Excluded | ✗ Excluded | ✗ Excluded | ✗ Excluded | ✓ Included ✗ | ✗ Excluded | ✓ Included ✗ | ✓ Included ✗ | ✓ Included ✗ | ✓ Included ✗ |
| **VA scaling** | /5 letters | Per-letter (Chat_1 categorical) | Per-letter (equivalent) | /5 letters | /5 letters | /5 letters | /5 letters | /5 letters | /5 letters | Per-letter (Python default) |
| **O1 cohort boundary** | VA <70 (strict) | VA <70 (5,794) | VA <70 (5,794) | VA <70 (5,978 ✓) | VA <70 (5,978 ✓) | VA <70 (5,794) | VA <70 (5,978 ✓) | VA <70 (5,978 ✓) | VA <70 (5,978 ✓) | VA ≤70 (5,809 ✗) |
| **O2 KM structure** | Simple Surv(tto70) | Simple Surv | Simple Surv | — | Simple Surv | Simple Surv | tmerge CP (wrong) | tmerge CP (wrong) | tmerge CP | tmerge CP |
| **Programming language** | R | R | R | R/Python | R/Python | R | R | R | R | Python |
| **Concordance available** | ✓ | ✓ (Chat_2/3) | ✓ (all 3) | ✗ (lifelines) | ✓ (inflated) | ✗ (lifelines) | ✗ | Partial | ✗ | ✓ (inflated) |
| **CP row count (O1)** | 35,949 | N/A | N/A | N/A | ~36,000 | N/A | ~60,600 (Chat_1) | 46,323 (Code_2) | ~36,000 | 83,088 ✗ |

This table summarizes the key structural implementation choices made by each run, independent of whether the resulting numerical outputs were accurate. Features are evaluated at the script Prompt By inspection.

✓ = implemented correctly; ✗ = deviation from reference.
CP = counting-process; VA = visual acuity; tmerge = survival::tmerge() in R.

## Table S5 — Full Quantitative Results: All 18 Prompt B, C Runs vs Reference

**Reference values:**

| **Outcome** | **Cohort n** | **KM Median (yr)** | **HR per 5 VA letters (95% CI)** | **Concordance** |
| --- | --- | --- | --- | --- |
| O1 (time to VA ≥70) | 5,978 | 2.04 | 1.434 (1.404–1.464) | 0.749 |
| O2 (duration VA ≥70) | 4,439 | 0.537 | 0.893 (0.880–0.905) | 0.598 |
| O3 (time to VA ≤35) | 6,453 | 8.66 | 0.704 (0.686–0.723) | 0.737 |

**Agent run results:**

| **Run** | **O1 n** | **O1 KM** | **O1 HR/5** | **O1 C** | **O2 n** | **O2 KM** | **O2 HR/5** | **O2 C** | **O3 n** | **O3 KM** | **O3 HR/5** | **O3 C** | **Notes** |
| --- | --- | --- | --- | --- | --- | --- | --- | --- | --- | --- | --- | --- | --- |
| B_Chat_1 | 5,794 | 2.03 | N/A | 0.700 | 4,396 | 0.537 | N/A | 0.551 | 6,269 | 8.65 | N/A | 0.716 | Script crash (categorical VA) |
| B_Chat_2 | 5,794 | 2.03 | 1.435 | 0.749 | 4,396 | 0.537 | 0.894 | 0.599 | 6,269 | 8.65 | 0.698 | 0.738 | — |
| B_Chat_3 | 5,794 | 2.03 | 1.435 | 0.749 | 4,238 | 0.537 | 0.894 | 0.597 | 6,269 | 8.65 | 0.698 | 0.737 | — |
| B_Code_1 | 5,794 | 2.03 | 1.435 | 0.749 | 4,238 | 0.537 | 0.894 | 0.597 | 6,269 | 8.65 | 0.698 | 0.737 | — |
| B_Code_2 | 5,794 | 2.03 | 1.435 | 0.749 | 4,238 | 0.537 | 0.894 | 0.597 | 6,269 | 8.65 | 0.698 | 0.737 | — |
| B_Code_3 | 5,794 | 2.03 | 1.435 | 0.749 | 4,238 | 0.537 | 0.894 | 0.597 | 6,269 | 8.65 | 0.698 | 0.737 | — |
| B_Cowork_1 | **5,978** | **2.034** | 1.427 | — | **4,439** | 0.539 | **0.893** | — | **6,453** | **8.657** | **0.705** | — | Python (lifelines); concordance not reported |
| B_Cowork_2 | **5,978** | **2.034** | **1.329** ✗ | 0.668 | **4,439** | 0.539 | **0.912** ✗ | 0.449 | **6,453** | **8.657** | **0.788** ✗ | 0.764 | injnum in Python Cox formula |
| B_Cowork_3 | 5,794 | 2.03 | 1.427 | — | 4,238 | 0.537 | **0.893** | — | 6,269 | 8.65 | **0.704** | — | Python (lifelines); concordance not reported |
| C_Chat_1 | **5,978** | **2.04** | **1.26** ✗ | — | 3,874 ✗ | — | — | — | **6,453** | 8.65 | — | — | O2 cohort construction error |
| C_Chat_2 | **5,978** | **2.04** | **1.219** ✗ | — | **4,439** | 0.537 | 0.891 | — | **6,453** | 8.654 | **0.772** ✗ | — | age ≥80 NA in Cox |
| C_Chat_3 | **5,978** | **2.04** | ~1.43 | — | 4,242 | 0.537 | ~0.893 | — | **6,453** | 8.66 | ~0.704 | — | — |
| C_Code_1 | **5,978** | **2.04** | **1.264** ✗ | — | **4,439** | 0.54 | **0.939** ✗ | — | **6,453** | 8.65 | **0.759** ✗ | — | Custom CP structure |
| C_Code_2 | **5,978** | **2.04** | 1.436 | 0.753 | **4,439** | 0.537 | 0.896 | 0.596 | **6,453** | 8.65 | **0.705** | 0.740 | — |
| C_Code_3 | **5,978** | **2.04** | 1.43 | — | **4,439** | 0.539 | **0.893** | — | **6,453** | 8.65 | **0.704** | — | — |
| C_Cowork_1 | **5,978** | **2.04** | 1.433 | — | **4,439** | 0.54 | 0.892 | — | **6,453** | 8.65 | **0.704** | — | — |
| C_Cowork_2 | 5,809 ✗ | 2.04 | **1.261** ✗ | **0.867** ✗ | 4,242 ✗ | 0.54 | — | **0.836** ✗ | 6,279 ✗ | 8.65 | **0.773** ✗ | 0.761 | Python CP; concordance inflated |
| C_Cowork_3 | — | — | — | — | — | — | — | — | — | — | — | — | Execution failure |

Bold = value matches reference (within ±0.05yr for KM; ±0.02 for HR/5). ✗ = outside threshold or structurally incorrect. — = not produced.
HR/5 = hazard ratio per 5 ETDRS letters; C = concordance statistic.
B_Cowork_1 and B_Cowork_3 used Python lifelines and did not report concordance statistics; values reported as "—".
C_Cowork_2 O1 concordance of 0.867 reflects counting-process interval inflation (83,088 rows vs reference 35,949 rows). See Method S4 for details.

## Table S6 — Proportional Hazard Diagnostics Comparison (Prompt B and C, 18 Runs)

| **Run ID** | **O1 Global χ^2 (p-val)** | **O2 Global χ^2 (p-val)** | **O3 Global χ^2 (p-val)** | **Findings vs. GT** |
| --- | --- | --- | --- | --- |
| **Ground Truth** | **150.39 (<0.001)** | **39.19 (<0.001)** | **74.63 (<0.001)** | Reference standard. |
| B_Chat_1 | 79.91 (<0.001) | 34.34 (0.0003) | 66.77 (<0.001) | Differs slightly due to categorical baseline VA modeling. |
| B_Chat_2 | 150.35 (<0.001) | 39.55 (0.00016) | 74.65 (<0.001) | Minor difference with correct cohort and analysis. The model omit ‘mean_inj_interval’ as covariate. |
| B_Chat_3 | 150.35 (<0.001) | 39.55 (0.00016) | 74.65 (<0.001) | Match |
| B_Code_1 | 150.35 (<0.001) | 39.53 (0.00016) | 74.65 (<0.001) | Match |
| B_Code_2 | 150.35 (<0.001) | 39.53 (0.00016) | 74.65 (<0.001) | Match |
| B_Code_3 | N/A | N/A | N/A | Did not perform analysis despite being asked |
| B_Cowork_1 | 150.35 (<0.001) | 39.53 (0.00016) | 74.65 (<0.001) | Match |
| B_Cowork_2 | 150.35 (<0.001) | 39.55 (0.00016) | 74.65 (<0.001) | Match |
| B_Cowork_3 | 150.35 (<0.001) | 39.53 (0.00016) | 74.65 (<0.001) | Match |
| C_Chat_1 | 857.08 (<0.001) | 42.69 (<0.001) | 224.30 (<0.001) | Replicated the original paper's multi-row start-stop counting-process format but omitted patient-level clustering. |
| C_Chat_2 | 857.08 (<0.001) | 42.69 (<0.001) | 224.30 (<0.001) | Same as C_Chat_1. |
| C_Chat_3 | 857.08 (<0.001) | 42.69 (<0.001) | 224.30 (<0.001) | Same as C_Chat_1. |
| C_Code_1 | 1065.22 (<0.001) | 1123.81 (<0.001) | 1266.34 (<0.001) | Constructed the multi-row dataset incorrectly by joining patient-level events directly to the longitudinal visit table without tmerge |
| C_Code_2 | 17.01 (0.1077) | N/A (tt() term) | 14.57 (0.2030) | **PH Violation Resolved** via stratification. O2 is N/A because cox.zph does not support time-transform tt() terms. |
| C_Code_3 | 17.01 (0.1077) | 31.52 (0.0028) | 14.57 (0.2030) | **PH Violation Resolved** via stratifying on va_inj1_group and loaded. |
| C_Cowork_1 | 857.08 (<0.001) | 42.69 (<0.001) | 224.30 (<0.001) | Same as C_Chat_1. |
| C_Cowork_2 | 150.35 (<0.001) | 39.55 (0.00016) | 74.65 (<0.001) | Match |
| C_Cowork_3 | 150.35 (<0.001) | 39.55 (0.00016) | 74.65 (<0.001) | Match |

## Table S7 — Script Output to Result Text Fidelity by Run

### Table S7A. Prompt B (9 Runs)

| **Run** | **Accurate to Log** | **Discrepancy Type** | **Notable Finding** |
| --- | --- | --- | --- |
| B_Chat_1 | ⚠ | Post-crash re-run undisclosed | Summary reflects corrected re-run, not crashing script; no disclosure |
| B_Chat_2 | ✓ | Unit propagation | HR 1.075/letter — non-standard unit propagates to narrative without conversion |
| B_Chat_3 | ✓ | Unit propagation | HR per letter propagated; partial TDC omission carried into summary |
| B_Code_1 | ✓ | Unit propagation | HR 1.075/letter correctly transcribed; all Code summaries nearly identical |
| B_Code_2 | ✓ | Unit propagation | Same as B_Code_1 |
| B_Code_3 | ✓ | Unit propagation | Same as B_Code_1 |
| B_Cowork_1 | ✓ | None | HR 1.43/5 letters (Python); clinically meaningful units; cohort correct |
| B_Cowork_2 | ✓ | Self-acknowledged bias | Biased HRs reproduced; explicit note compares biased vs corrected values |
| B_Cowork_3 | ✓ | None | Accurate to log |

### eTable7B. Prompt C (8 Completed Runs)

| **Run** | **Accurate to Log** | **Discrepancy Type** | **Notable Finding** |
| --- | --- | --- | --- |
| C_Chat_1 | ✓ | Code error transcribed | O2 cohort n=3,874 correct to log but reflects incorrect cohort definition; not hallucination |
| C_Chat_2 | ✓ | None | Biased HR 1.219 correctly reported |
| C_Chat_3 | ✓ | None | Accurate; well-structured |
| C_Code_1 | ✓ | None | Biased HRs accurately transcribed |
| C_Code_2 | ✓ | None | Near-reference values correctly reported |
| C_Code_3 | ✓ | None | Accurate; most detailed baseline table |
| C_Cowork_1 | ✓ | None | HR 1.433 and correct O2 direction reported |
| C_Cowork_2 | ✓ (to biased log) | Misleading clinical interpretation | Inverted O2 HR → "paradoxically shorter duration" — clinically incorrect but accurate to biased log |
| C_Cowork_3 | — | No output | — |

"Unit propagation" = non-standard HR unit (per letter vs per 5 letters) carried from code into narrative without clinical flagging. "Misleading clinical interpretation" = accurate to biased log; inverted and clinically incorrect interpretive statement.

## Table S8 — DECIDE-AI Reporting Guideline Checklist

*This study is an in silico evaluation of an LLM agent for data analysis, not a clinical deployment of a diagnostic decision support system. Items relating to live clinical users or prospective patient recruitment are marked Not Applicable (N/A) with rationale.*

| **Item No.** | **Theme** | **Reporting Recommendation** | **Reported on Page / Section** |
| --- | --- | --- | --- |
| **Title and abstract** |  |  |  |
| 1 | Title | Identify the study as early clinical evaluation of a decision support system based on AI or machine learning, specifying the problem addressed. | Title |
| I | Abstract | Provide a structured summary of the study. | Abstract |
| **Introduction** |  |  |  |
| 2a | Intended use | Describe the targeted medical condition(s) and problem(s), including the current standard practice, and the intended patient population(s). | Introduction |
| 2b | Intended use | Describe the intended users of the AI system, its planned integration in the care pathway, and the potential impact it is intended to have. | Introduction |
| II | Objectives | State the study objectives. | Introduction |
| **Methods** |  |  |  |
| III | Research governance | Provide a reference to any study protocol, study registration number, and ethics approval. | Methods (Evaluation Framework) |
| 3a | Participants | Describe how patients were recruited, stating the inclusion and exclusion criteria at both patient and data level. | Methods (Dataset and Clinical Context); Dataset from prior publication. |
| 3b | Participants | Describe how users were recruited, stating the inclusion and exclusion criteria. | N/A (In silico evaluation without live users) |
| 3c | Participants | Describe steps taken to familiarize the users with the AI system. | N/A (In silico evaluation without live users) |
| 4a | AI system | Briefly describe the AI system, specifying its version and type of underlying algorithm used. Describe the characteristics of the patient population on which the algorithm was trained. | Methods (AI Agent and System Prompting) |
| 4b | AI system | Identify the data used as inputs. Describe how the data were acquired, the process needed to enter the input data, and pre-processing applied. | Methods (Dataset and Clinical Context) |
| 4c | AI system | Describe the AI system outputs and how they were presented to the users. | Methods (AI Agent and System Prompting) |
| 5a | Implementation | Describe the settings in which the AI system was evaluated. | Methods (Experimental Conditions) |
| 5b | Implementation | Describe the clinical workflow/care pathway in which the AI system was evaluated, the timing of its use, and how the final supported decision was reached. | Methods (Evaluation Framework); Appendix eFigure 1 |
| IV | Outcomes | Specify the primary and secondary outcomes measured. | Methods (Evaluation Framework) |
| 6a | Safety and errors | Provide a description of how significant errors/malfunctions were defined and identified. | Methods (Evaluation Framework) |
| 6b | Safety and errors | Describe how any risks to patient safety or instances of harm were identified, analyzed, and minimized. | Methods (Evaluation Framework) |
| 7 | Human factors | Describe the human factors tools, methods or frameworks used, the use cases considered, and the users involved. | N/A (In silico analytical evaluation) |
| V | Analysis | Describe the statistical methods by which the primary and secondary outcomes were analyzed, including subgroup analyses. | Methods (Evaluation Framework) |
| 8 | Ethics | Describe whether specific methodologies were utilized to fulfil an ethics-related goal (such as algorithmic fairness). | Methods (Ethical considerations) |
| VI | Patient Involvement | State how patients were involved in any aspect of the study. | Methods (Ethical considerations) |
| **Results** |  |  |  |
| 9a | Participants | Describe the baseline characteristics of the patients included in the study, and report on input data missingness. | Appendix (Method S1); Original dataset publication |
| 9b | Participants | Describe the baseline characteristics of the users included in the study. | N/A |
| 10a | Implementation | Report on user exposure to the AI system, number of instances used, and user adherence to intended implementation. | Results (Run completion rates) |
| 10b | Implementation | Report any significant changes to the clinical workflow or care pathway caused by the AI system. | N/A (In silico study) |
| VII | Main results | Report on the pre-specified outcomes, including outcomes for any comparison group. | Results |
| VIII | Subgroups analysis | Report on the differences in the main outcomes according to pre-specified subgroups. | Results |
| 11 | Modifications | Report any changes made to the AI system or its hardware platform during the study. | N/A (Fixed model API used) |
| 12 | Human-computer agreement | Report on the user agreement with the AI system. | Results (Script Output to Result Text Fidelity) |
| 13a | Safety and errors | List any significant errors/malfunctions related to: AI system recommendations, supporting software/hardware, or users. | Results (Error Pattern Taxonomy, Table 3) |
| 13b | Safety and errors | Report on any risks to patient safety or observed instances of harm identified during the study. | Results (Error Pattern Taxonomy, Table 3) |
| 14 | Human factors | Report on usability evaluation and user learning curves. | N/A |
| **Discussion** |  |  |  |
| 15 | Support for intended use | Discuss whether the results obtained support the intended use of the AI system in clinical settings. | Discussion |
| 16 | Safety and errors | Discuss what the results indicate about the safety profile of the AI system, observed errors/malfunctions, and mitigation. | Discussion |
| IX | Strengths and limitations | Discuss the strengths and limitations of the study. | Discussion |
| **Statements** |  |  |  |
| 17 | Data availability | Disclose if and how data and relevant code are available. | Data Sharing Statement |
| X | Conflicts of interest | Disclose any relevant conflicts of interest, funding source, etc. | Conflict of Interest Disclosures |
